# Metabolic Reprogramming Induces Immune Cell Dysfunction in the Tumor Microenvironment of Multiple Myeloma

**DOI:** 10.3389/fonc.2020.591342

**Published:** 2021-01-15

**Authors:** Shaojie Wu, Huixian Kuang, Jin Ke, Manfei Pi, Dong-Hua Yang

**Affiliations:** ^1^Department of Hematology, Zhujiang Hospital, Southern Medical University, Guangzhou, China; ^2^Guangdong Key Laboratory of Orthopaedic Technology and Implant Materials, Medical Center of Assessment of Bone & Joint Diseases, Orthopaedic Hospital, General Hospital of Southern Theater Command, Guangzhou, China; ^3^College of Pharmacy and Health Sciences, St. John’s University, New York, NY, United States

**Keywords:** metabolic reprogramming, multiple myeloma, signaling pathways, tumor microenvironment, immune cell dysfunction

## Abstract

Tumor cells rewire metabolism to meet their increased nutritional demands, allowing the maintenance of tumor survival, proliferation, and expansion. Enhancement of glycolysis and glutaminolysis is identified in most, if not all cancers, including multiple myeloma (MM), which interacts with a hypoxic, acidic, and nutritionally deficient tumor microenvironment (TME). In this review, we discuss the metabolic changes including generation, depletion or accumulation of metabolites and signaling pathways, as well as their relationship with the TME in MM cells. Moreover, we describe the crosstalk among metabolism, TME, and changing function of immune cells during cancer progression. The overlapping metabolic phenotype between MM and immune cells is discussed. In this sense, targeting metabolism of MM cells is a promising therapeutic approach. We propose that it is important to define the metabolic signatures that may regulate the function of immune cells in TME in order to improve the response to immunotherapy.

## Introduction

Multiple myeloma (MM) is a hematologic malignant proliferative disease characterized by the unrestricted proliferation of plasma cells in the bone marrow with overproduction of monoclonal immunoglobulin or light chain proteins (1). Although the overall survival of MM patients has significantly improved in the recent years, this disease is still incurable (2). Treatment options are limited especially for refractory and recurrent patients who have been treated with proteasome inhibitors, immunomodulatory drugs, as well as monoclonal antibodies. Immunotherapies, including oncolytic vaccines, checkpoint inhibitors, and adoptive cellular immunotherapy, offer a potentially effective treatment to these patients. However, the therapeutic efficacy is limited to a small number of population. Thus, it is urgent to look for alternative therapies.

Metabolic reprogramming is considered a hallmark of cancers and metabolism is altered in most, if not all cancer cells, regardless of the type of cancers, to meet the needs of energy and biosynthesis for rapid cell proliferation, and to adapt to the tumor microenvironment (TME). Glycolysis and glutaminolysis enhancement are two of the most common but vital modalities in TME (3, 4). Recent studies have revealed that metabolic reprogramming may affect the TME and, more importantly, could impact the function of immune cells. Therefore, some researchers suggested that the treatment response of MM might depend, at least in part, on the function of immune cells and tumor cell metabolic status in the TME (5).

In this review, we discuss the metabolic reprogramming in MM, which is associated with a hypoxic, acidic, and nutritionally deficient TME, and how these changes impede the function of immune cells. Metabolic changes of glucose and glutamine are described in detail. We suggest that integration of targeting tumor metabolism with immunotherapy could be a rational approach to modulate the TME and improve therapeutic efficacy of MM.

## Metabolic Changes in MM Cells

**Figure 1:**
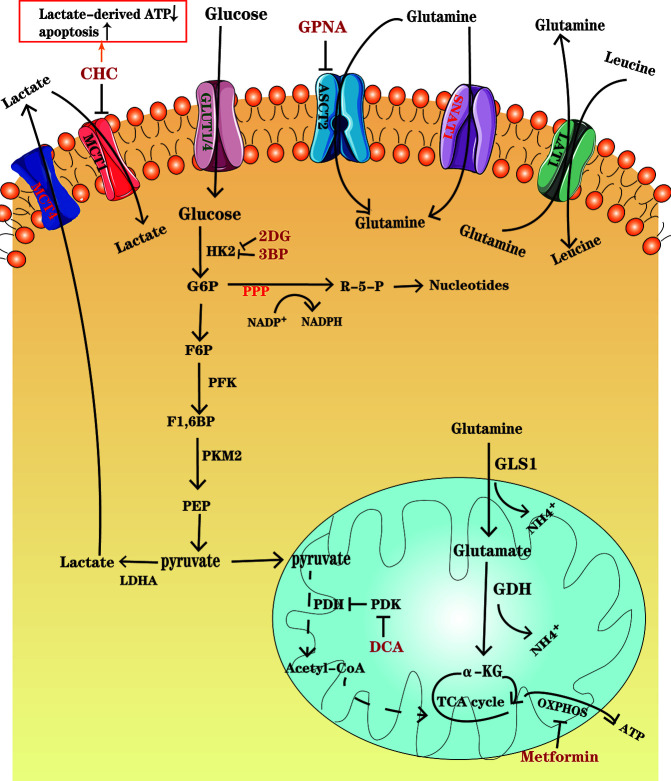
An overview of glucose and glutamine metabolism in MM cells. Glucose enters MM cells through GLUT1 and GLUT4 and is transformed into lactate depending on several key enzymes, including HK2, PFK, PMK2, and LDHA. Lactate is transported out of the cells through MCT4. Some cancer cells in normoxic areas may exploit lactate secreted from hypoxic cancer cells as an energy source and import it into cells by MCT1. PDK, the PDH inhibitor, is overexpressed in MM ells, which results in a decrease in pyruvate entering TCA cycle. Glutamine, as the major substrate for TCA cycle intermediates, enters the MM cells through transporters ASCT2 and SNAT1, in which ASCT2 is the major type. Glutamine can be exported out of the cell through antiporters LAT1 in exchange for other amino acids such as leucine. Glutamine is converted to glutamate by the enzyme GLS and further to a-KG by GDH, which fuel the TCA cycle. Drugs, such as CHC, GPNA, 2DG and so on, have been designed to inhibit the important enzymes and transporters and thus to disturb metabolism in MM cells. Further details are found in the main body of text.

In contrast to the reliance on oxidative phosphorylation (OXPHOS) to obtain energy in normal cells, most cancer cells adapted to their microenvironment rely heavily on aerobic glycolysis, converting glucose into lactic acid, for rapid production of adenosine triphosphate (ATP) to provide competitive advantages to cancer cells, thus meeting the requirements of rapid division and growth. This phenomenon is known as the Warburg effect (6), which is a hallmark of cancers, and is utilized in the 18F-fluorodeoxyglucose PET (18F- FDG–PET) as a sensitive diagnostic and prognostic tool in clinic (7, 8). MM is also reported to be dependent on glycolysis due to an elevated glycolytic gene profile, as well as its susceptibility to glycolysis inhibitors, such as inhibitors of glucose transporter (GLUT) and key glycolytic enzyme (9). The GLUT family comprises of 14 GLUT subtypes (10), among which GLUT1 overexpression is associated with poor clinical outcomes in various cancers. Researchers have found that GLUT1 up-regulation enables MM cells to elevate glucose uptake and GLUT1-specific inhibition can selectively induce death in MM cells with high GLUT1 level (11). However, other study has demonstrated that MM cells rely on GLUT4 for fundamental glucose uptake, maintenance of growth, survival and Mcl-1 expression (12). After glucose is transported into the cells, it is transformed into lactate and produces ATP through a multi-step metabolism depending on several key enzymes, including hexokinase 2 (HK2), phosphofructokinase (PFK), pyruvate kinase M2 (PKM2) and lactate dehydrogenase A (LDHA) that are highly expressed in MM (13) (**Figure 1**). Aerobic glycolysis enhancement also activates the pentose phosphate bypass pathway (PPP) and leads to increased production of reduced nicotinamide adenine dinucleotide phosphate (NADPH) and glutathione (GSH), both benefit tumor cells against oxidative damage (14). Since oxidative stress is one of the important mechanisms of using bortezomib, drug resistance may be accompanied by increased antioxidant capacity. GP Soriano et al. have proposed that the generation of NADPH makes MM cells more tolerant to proteasome inhibitors (15). Lactate, as a glycolysis product, is transported by monocarboxylate transporters (MCTs) MCT1 and MCT4. Several studies have indicated that lactate can be used as a fuel for OXPHOS (16, 17). Fujiwara et al. found that MCT1 expression, which preferentially promotes lactate import, is up-regulated in MM cells under aerobic conditions, while MCT4, which favors lactate export, is upregulated under hypoxic conditions (18). It has been suggested that lactate secreted from hypoxic cancer cells may be used by normoxic cancer cells as an energy source since it can be converted to pyruvate, and then enter the tricarboxylic acid (TCA) cycle, establishing a metabolic symbiosis between hypoxic and normoxic cancer cells, which is important for the fast-growth and progression of tumors (19). Sevim et al. have proved that MM cells are capable of enhancing mitochondrial OXPHOS under ritonavir treatment (20). Subsequently, Christopher et al. have reported that increased OXPHOS in MM cells is associated with CD38-driven mitochondrial transfer (21).

Studies using myeloma cell lines have also shown the significance of glutamine in plasma cell metabolism. The growth of MM cells is limited by glutamine depletion (22). Glutamine, which is an important nitrogen donor for synthesis of amino acids and nucleotides, and the major substrate for TCA cycle intermediates, is indispensable to vigorous cell proliferation (23). Bortezomib-resistant cell lines have shown enhanced mitochondrial function fueled by glutamine rather than glucose. Therefore, interfering with glutamine metabolism has great potential in the treatment and overcoming drug resistance in MM (24). c-Myc is an important factor, which contributes to the tumorigenic phenotype of myeloma cells and increases glutamine transporters and glutaminase (GLS) expression to favor glutaminolysis (25). In glutaminolysis, glutamine, transported into the cells through transporters such as SLC1A5 (also known as ASCT2) and SNAT1 (26), is converted to glutamate by the enzyme GLS and further to α-ketoglutarate (α-KG) by glutamate dehydrogenase (GDH), which fuels the TCA cycle (27, 28) (**Figure 1**). A study has demonstrated that in the development of monoclonal immunoglobulin disease, CD138^+^ cells enhance the expression of glutamine transporters ASCT2, LAT1 and SNAT1. However, only ASCT2 inhibition in human myeloma cell lines (HMCLs) causes a marked decrease in glutamine uptake and cell growth (29). It has also been shown that HMCLs express GLS1 and are sensitive to its inhibition, whereas they negligibly express glutamine synthetase (GS) and GLS2 (29), which demonstrates that MM cells mainly depend on extracellular glutamine uptake.

In summary, myeloma cells appear to be more dependent on glycolysis and glutaminolysis than normal plasma cells. Therefore, targeting glycolysis and glutaminolysis could be used for the treatment of the disease. However, the compensatory enhancement of mitochondrial OXPHOS when glycolysis is inhibited in MM should also be noted.

## Metabolic Changes in Immune Cells

While it is well known that cancer cells undergo metabolic reprogramming, as described above, there is a growing recognition that such metabolic alterations also occur in immune cells, which affects the function of immune cells and contribute to tumor immune escape (30–32).

The increasing emergence of evidence implies that polarized macrophages exhibit different metabolic patterns. M1 macrophages induce increased anabolic metabolism, such as glycolysis, PPP and fatty acid biosynthesis (FAS), whereas M2 macrophages induce OXPHOS. Specifically, aerobic glycolysis is promoted by toll like receptor (TLR) -induced signaling, which stabilizes hypoxia-inducible factor (HIF)-1α and boosts mammalian target of rapamycin (mTOR) activity (33). Similarly, T cells use distinct metabolic pathways in the process of activation and differentiation. Regarded as quiescent population, naïve and memory T cells mainly rely on OXPHOS and fatty acid β-oxidation (FAO) to produce ATP and show reduced rates of nutrient uptake and biosynthesis (34, 35). However, after activation, T-effector cells rapidly switch towards glycolysis and glutaminolysis to produce ATP and metabolic resources rapidly and meet the demand for daughter cell generation and suppress FAO (36). This change is orchestrated by T cell receptor (TCR) and CD28, which activate the phosphatidylinositol 3’-kinase (PI3K) -AKT-mTOR pathway and the transcription factors HIF-1α and c-myc, thus contributing to the up-regulation of metabolic enzymes, glucose and amino acid transporters (37). Likewise, activated NK cells also boost glycolysis (38). PI3K is required for multiple key aspects of NK cell biology, including maturation, homing, priming, and functioning (39).

Interestingly, some types of anti-tumor immune cells share many metabolic needs with MM cells, which leads to an energy inter-dependence that may cause metabolic competition between them. In general, cancer cells have better access to nutrients than immune cells, inducing hypoxic and acidic areas with nutrient depletion and lactate accumulation, thus promoting the development and spread of tumors and suppressing immune surveillance (40). For example, the lack of glucose and glutamine impairs TCR signaling, and is harmful for glycolysis and anti-tumor functions of T-effector cells. However, regulatory T (Treg) cells, preferring FAO to glycolysis, are able to survive under these conditions and act as immunosuppressive cells. Actually, the activation of AMP-activated protein kinase (AMPK), a sensor of nutrient stress, correlates with Treg cells expansion (41). Although the specific mechanism of metabolic switch in immune cells during MM development has not been thoroughly studied, this competition has led to the hypothesis that controlling the metabolism of MM cells may inhibit their growth by not only killing them directly, but also restoring the function of immune cells indirectly.

Autologous stem cell transplant (ASCT) and other novel anti-MM therapies have emerged in recent years. Such treatments have changed the composition and metabolic profile of MM cells and immune cells, particularly T cells. Compared with healthy donors, immune cells in relapsed/refractory MM (RRMM) patients increase FAO and mitochondrial respiration (42). This might result from MM cells creating a microenvironment of chronic inflammation in the bone marrow, particularly the increased production of IL-6, which drives the metabolic alteration of immune cells (43, 44). Anti-MM therapies and ASCT might disrupt the composition of immune cell populations and promote metabolic rewiring. In the TME, there are a significant reduction of CD4^+^ naïve T cells, an increase in CD8^+^ memory T cells and an increase in CD4^+^ T cells that overexpressed PD1, that might perturb the fitness in immunotherapies, particularly chimeric antigen receptor (CAR)-T therapy. Understanding these changes might guide ASCT and other immune therapies for MM. A study suggested that storing up T cells before ASCT rather than at relapse may offer a more effective CAR-T therapy (42).

## Molecular Signaling Pathways and Transcription Factors Leading to Metabolic Reprogramming in MM

**Figure 2:**
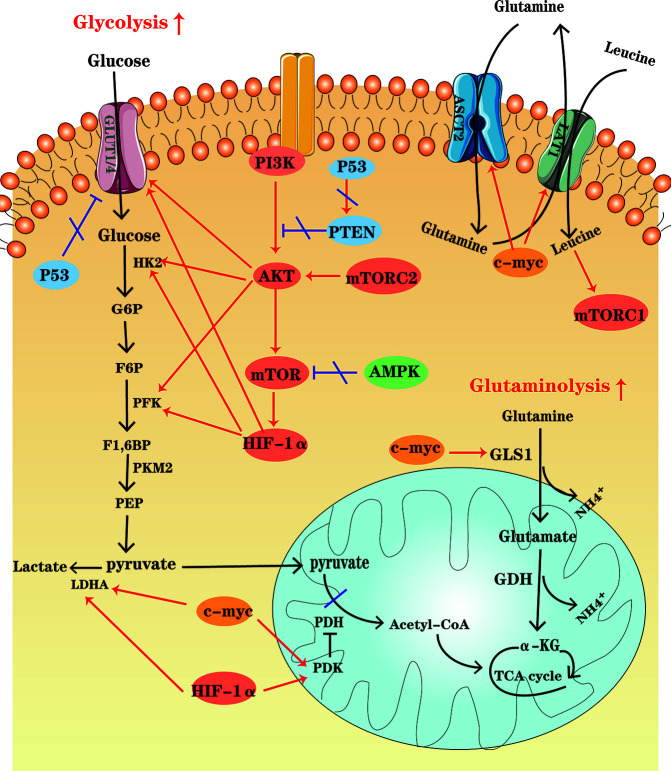
An overview of cellular metabolic pathways in MM cells. In MM cells, PI3K-AKT-mTOR pathway up-regulation, the high HIF-1 expression and the increased c-myc activity enhance glycolysis and glutaminolysis, which foster expression of key enzymes and transporters. At the same time, AMPK down-regulation, as well as P53 deficiency contribute to the metabolic reprogramming of MM cells in the glycolytic direction.

As explained above, MM and immune cells show complex metamorphosis in metabolism in the course of tumor progression. Below, we discuss a few crucial molecular signaling pathways and transcription factors that regulate metabolism in MM (**Figure 2**).

### PI3K-AKT Pathway

The PI3K-Akt signaling pathway is activated by a variety of cellular stimuli, to regulate transcription, translation, proliferation, growth, survival and other basic cell functions. It has been found that PI3K-Akt signaling pathway is upregulated in MM, which is activated by some key cytokines associated with MM pathogenesis, such as interleukin (IL)-6 (45, 46) and stromal-derived factor (SDF)-1 (47, 48). Once it is activated, Akt can promote glycolysis by activating several glycolytic enzymes, including HK and PFK, and upregulating GLUTs (49). Akt is also a major trigger of mTOR activation. mTOR, the serine/threonine kinase, consists of mTORC1 and mTORC2 complexes. mTORC1 can drive the expression of several glycolytic enzymes such as PFK by increasing HIF-1α translation, promoting a shift in glucose metabolism from OXPHOS to glycolysis. The most important role of mTORC2 is likely to foster Akt phosphorylation and activation (50).

### AMPK Pathway

Expressed in essentially all eukaryotic cells, AMPK is a sensor of cellular energy status. When the ratios of AMP : ATP and ADP : ATP increase, it indicates cellular energy stress and positively regulates signal transduction pathways that generate ATP, FAO and autophagy, and to negatively regulates ATP-consuming biosynthesis processes, including gluconeogenesis, FAS, and protein synthesis (51). Moreover, contributing to direct phosphorylation and activation of tuberous sclerosis complex 2 (TSC2), AMPK activation results in suppression of the mTOR signaling pathway (50). Tumor cells seem to downregulate AMPK under selective pressure, thereby limiting its inhibitory effect on cell growth and proliferation (52). Studies have shown that up-regulation of the AMPK signaling pathway may be beneficial for MM treatment. For instance, metformin can inhibit mitochondrial complex I activity, and thus cause an increase in AMP : ATP and ADP : ATP ratios, which activate AMPK indirectly and inhibit mTOR signaling, decreasing IL-6R expression (46).

### Transcription Factors: HIF-1α, c-MYC, and P53

Composed of an inducibly expressed HIF-1α subunit and a constitutively expressed HIF-1β subunit, HIF-1 is a transcription factor known as a major regulator of hypoxia, which is a common condition in TME. Previous studies have shown that hypoxia is a characteristic of the specific bone marrow niche of MM and regions of hypoxia develop during MM progression (53, 54). Immunohistochemical staining has shown that HIF-1α is highly expressed in MM bone marrow. HIF-1α is an important regulator of cellular metabolism (55). HIF-1α induces the expression of glycolytic genes, including GLUT1, HK2, PFKFB3, LDHA, and pyruvate dehydrogenase kinase (PDK), and TCA cycle suppressors (56, 57). Inhibition of HIF-1α enables drug resistant MM cells to restore sensitivity (58, 59).

Members of the *MYC* family are important oncogenes involved in the development of malignant cells, and *c-MYC* activity is enhanced in MM (60). Actually, *MYC* regulates all genes involved in glycolysis and most genes in glutaminolysis (61). For instance, combining with HIF, c-MYC induces the expression of glucose transporters and enzymes such as LDHA to enhance glycolysis and PDK1 to inhibit pyruvate dehydrogenase (PDH) activity, thereby impairing mitochondrial function. Besides, c-MYC plays a significant role in inducing the major transporter and enzyme expression for cancer cell glutamine metabolism, including ASCT2 and GLS1 (4, 61).

The tumor suppressor P53 is altered in approximately 50% of human cancers (62). The aberrant *TP53* gene, resulted from the deletion or mutation of the *TP53* gene (TP53mut), is one of the key biomarkers of poor prognosis of MM (63). P53 represses glycolysis and thus favors OXPHOS by downregulating SLC2A1/4, which encodes the glucose transporters GLUT1 and GLUT4, and upregulating PTEN, a tumor suppressor gene, which inhibits PI3K-Akt pathway. In other words, P53 defect contributes to the metabolic rewiring of cancer cells in a more glycolytic direction (64).

## The Impact of Cellular Metabolic Changes on the TME and Immunosurveillance

**Figure 3:**
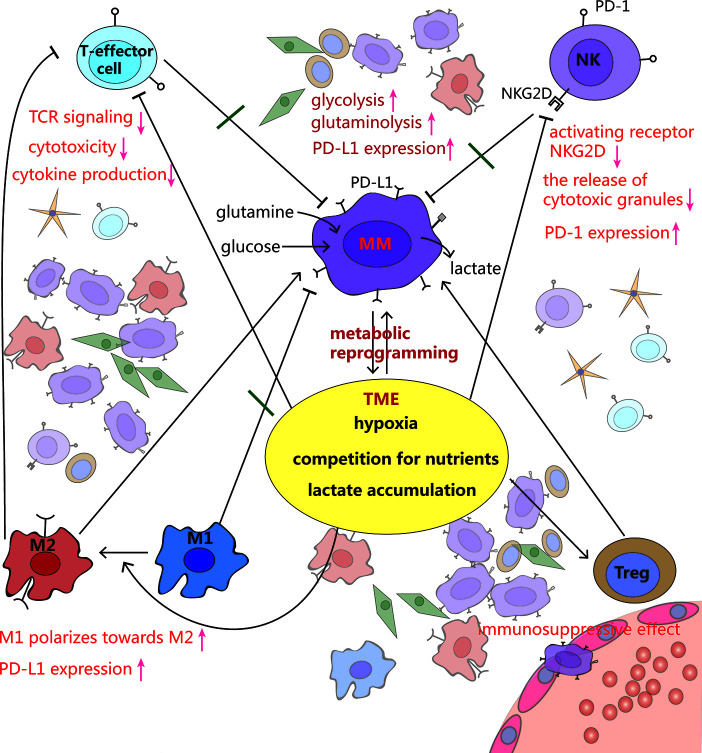
Crosstalk among MM cells, immune cells and tumor microenvironment (TME). MM cells rewire metabolism, such as increased glycolysis and glutaminolysis, to adapt to the hypoxic TME. Meanwhile, this makes the TME more anoxic, acidic and less nutritious, which inhibits T effector cells and NK cells, stimulates polarization from pro-inflammatory M1 macrophages toward a cancer-promoting M2 phenotype, as well as promotes Tregs development, leading to immune evasion.

The growth of MM requires a large amount of oxygen and nutrients, and produces a lot of lactate. This metabolic shift shapes the TME towards a hypoxic, acidic, and nutritionally deficient one, which supports cancer proliferation and metastasis (65, 66). However such TME is extremely unfavorable for immune cells to exert their antitumor effects (5, 31, 67) (**Figure 3**).

### Hypoxia

Since tumor aerobic glycolysis consumes a large amount of oxygen, the TME is often anoxic. Studies have indicated that overwhelming anti-tumor immunity is mostly due to hypoxia in the TME (68, 69).

Ikeda et al. have demonstrated that under low oxygen conditions, miR-210 is activated in MM cells (70). Remarkably, Noman et al. have shown that high level of miR-210, regulated by hypoxia, significantly blunts the susceptibility of tumor cell to cytotoxic T cell-mediated lysis by silencing of PTPN1, HOXA1, and TP53I11 in melanoma and lung cancer (71). Among them, PTPN1 and TP53I11 were found dramatically downregulated in MM (72).

NK cells have unique recognition function and cytotoxic function, that play crucial roles in immune monitoring and fighting against cancer cells (73). The receptors NKG2D and DNAM-1 are required for NK cell-mediated killing by identifying ligands RAE-1 and PVR expressed on MM cells, respectively (74, 75). However, the expression of these receptors decreases on NK cells derived from MM patients, resulting in impairment of NK cell function (76, 77). Several studies have suggested that hypoxia decreases NKG2D expression on NK cell surface, partially by hypoxic-tumor-derived micro-vesicles expressing immune modulatory factor TGF-β (78, 79). Similarly, NKG2D ligand-bearing micro-vesicles interfere with the function of NK cells, which kills cancer cells in a NKG2D-dependent manner (78).

Hypoxia, *via* HIF-1α, directly upregulates PD-L1 expression in various tumor cells by directly binding to the HRE in PD-L1 gene promoter, causing immunosuppressive TME (51, 80). The expression of PD-L1 on plasma cells (PCs) has been reported to be higher in MM patients than those of MGUS patients. Compared to PCs from healthy donors, PD-L1 expression on cells from minimal residual disease (MRD) positive MM patients is also upregulated (81, 82). Additionally, NK cells, derived from patients with MM, express PD-1 whereas normal NK cells do not. PD-1 inhibits NK cell cytotoxicity through the engagement of PD-L1/PD-1 pathway (83).

### Lactate Accumulation

As a result of enhanced glycolysis, high concentration of lactate exerts its immunosuppressive function by suppressing lymphocyte proliferation, cytokine production and cytotoxic activity (36, 84). Studies have reported that tumor cell–derived lactate, which lowers the pH of TME to values below 6-6.5, is able to render T lymphocytes anergic due to reduced cytokine secretion including IFN-γ, IL-2, and TNF-α, decreased expression of TCR and IL-2 receptor CD25, as well as impairment of STAT5 and ERK activation after TCR binding. Buffering the pH to a physiologic level can reverse T cell anergy (66, 85).

Macrophages have great plasticity and exhibit different phenotypes when stimulated by the environment. The number of macrophages increased in the BMM of MM patients and support MM cell proliferation and survival through contact- dependent and –independent pro-proliferative molecule STAT3 activation (86). Mediated by G protein-coupled receptors (GPCRs), that sense the tumor acidic environment, macrophages express inducible cyclic AMP early repressor (ICER), which inhibits TLR-dependent NF-κB signaling, and thus prevents macrophages from polarizing toward a pro-inflammatory phenotype (87). Recently, Chen et al. (88) found that lactic acid can be linked to histones in the macrophage genome in the form of epigenetic modification of lactylation, regulating the switches of relevant genes, and promoting the transformation of macrophages from the pro-inflammatory and anticancer M1 type to the anti-inflammatory and cancer-promoting M2 type. Expressed lactate-induced ligands PD-L1, these M2-like tumor-associated macrophages (TAMs) also can blunt effector T cell function (89).

## Targeting Metabolic Vulnerabilities in MM

As explained in the previous sections of this review, the metabolic pattern of MM cells is different from that of normal PCs, due to its eagerness for nutrients, especially glucose and glutamine. Importantly, the metabolic competition between MM and immune cells, together with the TME may exert an unfavorable effect on the functions of anti-tumor immune cells. Therefore, targeting cell metabolism by making use of the obvious distinctions between MM and normal cells, or integrating MM cell metabolism manipulation with immunotherapy seems to be an attractive anticancer strategy. However, the overlapping metabolic requirements between MM and immune cells should be taken into consideration.

Glucose metabolism is the focus of cancer metabolism research, including MM. Therefore, drugs have been designed to target glycolysis for cancer treatment. Most of them are proposed to inhibit key glycolytic enzymes (**Figure 1**).

HK2, the first interesting enzyme that catalyze glycolysis, is highly expressed in MM cells and is the most important target of 3-bromopyruvate (3BP), the small alkylating compound. Entering cells through MCTs, 3BP utilizes the distinct differences in MCT expression between tumor and normal cells, making cancer cells susceptible to it and decreasing the harm to normal cells with a proper concentration (90). In a few cancer researches, 3BP has been shown to be strikingly effective because of its multiple targets, enabling it to inhibit both glycolysis and mitochondrial OXPHOS (91). In addition to disruption of ATP production, 3BP can cause a remarkable decrease of reduced GSH level, as well as induce cell apoptosis, that were confirmed on MM cells (92).

2-Deoxyglucose (2DG), a glucose analog, is phosphorylated by HK2 and thus form a non-metabolizable substance, 2-DG-6-phosphate after entering cancer cells, which interferes with glycolysis (13). However, 2DG alone has not been reported to eradicate cancers in animals quickly. It has also been shown to decrease the secretion of cytokines and compromise the effector functions of T cells (93). In contrast, 2DG used to inhibit glycolysis has been demonstrated to favor the generation of memory T cells, whose number within tumors relates to a better overall survival (94–96). However, it has not been fully studied in MM. According to the current evidence, 3BP may has a better therapeutic effect compared to 2DG in MM.

Similar to HK2, PKM2 is another important enzyme of glycolysis. Mitosis gene A (NIMA)-related kinase 2 (NEK2), transcriptionally modulated by c-MYC in MM cells, can promote aerobic glycolysis by regulating PKM splicing and increasing the PKM2/PKM1 ratio. Myeloma patients with NEK2 and PKM2 overexpression have reduced event-free and overall survival (97). It has recently been revealed that P5091,a nonreversible USP7 inhibitor, can deplete NEK2 protein *in vitro* and *in vivo*, resulting in suppression of NEK2 downstream target, thus overcoming bortezomib resistance (98).

As a fuel for OXPHOS, lactate is incorporate into MM cells by MCT1. Inhibition of MCT1 by its inhibitor, such as α-cyano-4-hydroxycinnamic acid (CHC), or by gene-silencing technique, has proved to reduce lactate incorporation and lactate-derived ATP production significantly, as well as induce MM cell apoptosis. Additionally, the combination of CHC with dichloroacetate (DCA), a PDK inhibitor, can enhance MM cell death. DCA has also additive cytotoxicity when used in combination with bortezomib (16, 99). However, it promotes the formation of Treg cell (100).

Previous studies have demonstrated that inhibition of GLUT4 leads to apoptosis and cell arrest in MM cells. However, a portion of MM cells survive with medication targeting glycolysis, likely through the compensatory enhancement of mitochondrial OXPHOS (20). Metformin, universally described as an antihyperglycemic drug, can inhibit the activity of mitochondrial complex I (**Figure 1**), activate AMPK pathway, as well as downregulate mTOR (101), which induce MM cell autophagy and cell cycle arrest in G0/G1, rather than cell apoptosis (102). Combination of the GLUT4 inhibitor ritonavir with the complex I inhibitor metformin, which increases dependence on glycolytic ATP production, has been shown to overcome ritonavir resistance and effectively elicit cell apoptosis in both *in vivo* and vitro studies of MM (20). Additionally, metformin enhances cytotoxic T cell function through phosphorylation of PD-L1 at S195, which induces PD-L1 glycosylation and degradation in a breast cancer model (103).

Glutaminolysis enhancement is another metabolic change observed in MM, which is important for maintenance of energy production and redox control (28). A subset of cancer cells are resistance to treatment of glycometabolism inhibition, which may result from compensatory increased glutamine consumption, suggesting that inhibition of both glutaminolysis and glycolysis might be a feasible therapy (40).

Glutamine transporter ASCT2 can be inhibited by l-γ-glutamyl-p-nitroanilide (GPNA), thus decreasing glutamine uptake and suppressing cancer cell proliferation (104). As mentioned before, GS expression in MM cells is absent, whereas GLS expression is elevated. It has been shown that GLS inhibitor CB-839 in combination with proteasome inhibitors carfilzomib exerts a synergistic cytotoxic effect (105). Recently, Powell et al. (106) found that the compound JHU083, which blocks glutamine metabolism can disrupt tumor metabolism, paralyze the “Warburg effect” of tumors, reverse hypoxia, polyacid, and nutrient deficiency in the TME, and relieve the immunosuppression ability of tumor microenvironment. In addition, this small molecule can also reprogram the metabolism of T cells, directly activating T cells, increasing their longevity and promoting memory T cells formation. The researchers have named this treatment as “metabolic checkpoint” inhibitors. Since Powell et al. have studied only four cancer cell lines, colon cancer MC38, lymphoma el-4, colon cancer CT26, and melanoma B16, till date, MM treatment remains to be explored.

## Conclusion

MM is an extremely heterogeneous and complex disease and is incurable in spite of great advances in therapeutic strategies. Numerous studies on metabolic reprogramming have revealed various targets in the process of glucose and glutamine metabolism. Metabolic reprogramming in MM creates a hypoxic, acidic, and nutritionally deficient TME that inhibits normal cell growth and immune cell function. The overlapping metabolic phenotype between MM and immune cells must be identified. Targeting metabolic pathways and promoting the function of immune cells could be a novel strategy for the cure of MM.

## Author Contributions

SW and D-HY formulated the topic of the review. SW, HK, and JK drafted the manuscript. D-HY edited the manuscript. All authors contributed to the article and approved the submitted version.

## Funding

This work was supported by the National Natural Science Foundation of China (81400156).

## Conflict of Interest

The authors declare that the research was conducted in the absence of any commercial or financial relationships that could be construed as a potential conflict of interest.

## Abbreviations

G6P, glucose-6-phosphate; F6P, fructose-6-phosphate; F1,6BP, fructose-1,6-biphosphate; PEP, phosphoenolpyruvate; TGF-β, transforming growth factor beta; PD-1, programmed cell death-1; PD-L1, programmed cell death-ligand 1.
